# First reported case in Canada of anaphylaxis to lupine in a child with peanut allergy

**DOI:** 10.1186/s13223-018-0303-4

**Published:** 2018-10-29

**Authors:** Lianne Soller, Sebastien La Vieille, Edmond S. Chan

**Affiliations:** 1 Division of Allergy and Immunology, Department of Pediatrics, Faculty of Medicine, University of British Columbia, 4480 Oak St, Rm 1C11, Vancouver, BC V6H 3V4 Canada; 2 Bureau of Chemical Safety, Food Directorate, Health Canada, 251 Sir Frederick Banting Driveway, Ottawa, ON K1A 0K9 Canada; 3 Division of Allergy and Immunology, Department of Pediatrics, Faculty of Medicine, University of British Columbia, 4480 Oak St, Rm 1C31B, Vancouver, BC V6H 3V4 Canada

**Keywords:** Lupine, Lupine allergy, Peanut allergy, Emerging allergen, Legume, Food allergy

## Abstract

**Background:**

Lupine is a member of the legume family and is often used in many food products in Europe (e.g. pasta, pizza, sauces, etc.) as a wheat or soy substitute. Lupine cross-reacts with peanut, and cases of allergic reactions to lupine in peanut-allergic patients have been reported in Europe mainly. In contrast, lupine as an ingredient in food products is relatively new to the Canadian market.

**Case presentation:**

We describe a 10-year old boy with diagnosed peanut and tree-nut allergy, who developed anaphylaxis to lupine flour in May 2017. A few minutes after eating a pre-made pancake mix that didn’t contain any of his known allergens (peanuts, tree nuts), he developed oral pruritis followed by throat tightness, severe stomach ache, lightheadedness, cough, hoarse throat, nasal congestion, sneezing, and fatigue. He refused epinephrine, but was given cetirizine. The symptoms resolved after 3 h, but he was still unwell the following day. In a conversation between the mother and the allergist, it was determined that lupine was likely the cause of the reaction. To confirm, he was brought into clinic for skin testing to lupine. Results were consistent with lupine allergy (pancake mix: 10 × 7 mm, lupine bean: 12 × 6 mm). The family has since reported this to the Canadian Food Inspection Agency, resulting in a product recall and a consumer advisory bulletin published by Health Canada.

**Conclusions:**

This is the first reported case of allergic reaction to lupine in Canada, and highlights the need for education of Canadian families with peanut allergy as well as allergists, regarding the possibility of cross-reactivity between peanut and lupine and its new presence in the Canadian food supply. In addition, a precautionary label for those with peanut allergy who purchase products containing lupine should be considered. This case illustrates also the need for a clear mechanism for consumers and allergists to report emerging food allergens to regulatory bodies such as Health Canada.

**Electronic supplementary material:**

The online version of this article (10.1186/s13223-018-0303-4) contains supplementary material, which is available to authorized users.

## Background

Lupine is a member of the legume family and is increasingly used in Europe in baked goods as an alternative to wheat/soy flour due to it being gluten-free and its health benefits (high protein, high fibre, low fat content) [1, 2]. Lupine is recognized as a priority allergen in Europe, Australia, and New Zealand, due to the severity of the allergic reactions reported, notably in peanut-allergic individuals, and its increased use as a food ingredient. Studies have shown that approximately 15–20% of peanut-allergic individuals are sensitized to lupine [3, 4]. In one study in France, 28% of peanut-allergic patients reacted to lupine at the same dose during an oral challenge [5], whereas a study in the UK found a 4% cross-reactivity [6]. The first report of lupine allergy in 1994 involved a 5-year old girl with known peanut allergy who experienced an allergic reaction after eating pasta with lupine flour [7]. Lupine allergy has not previously been reported in Canada.

## Case presentation

**Fig. 1:**
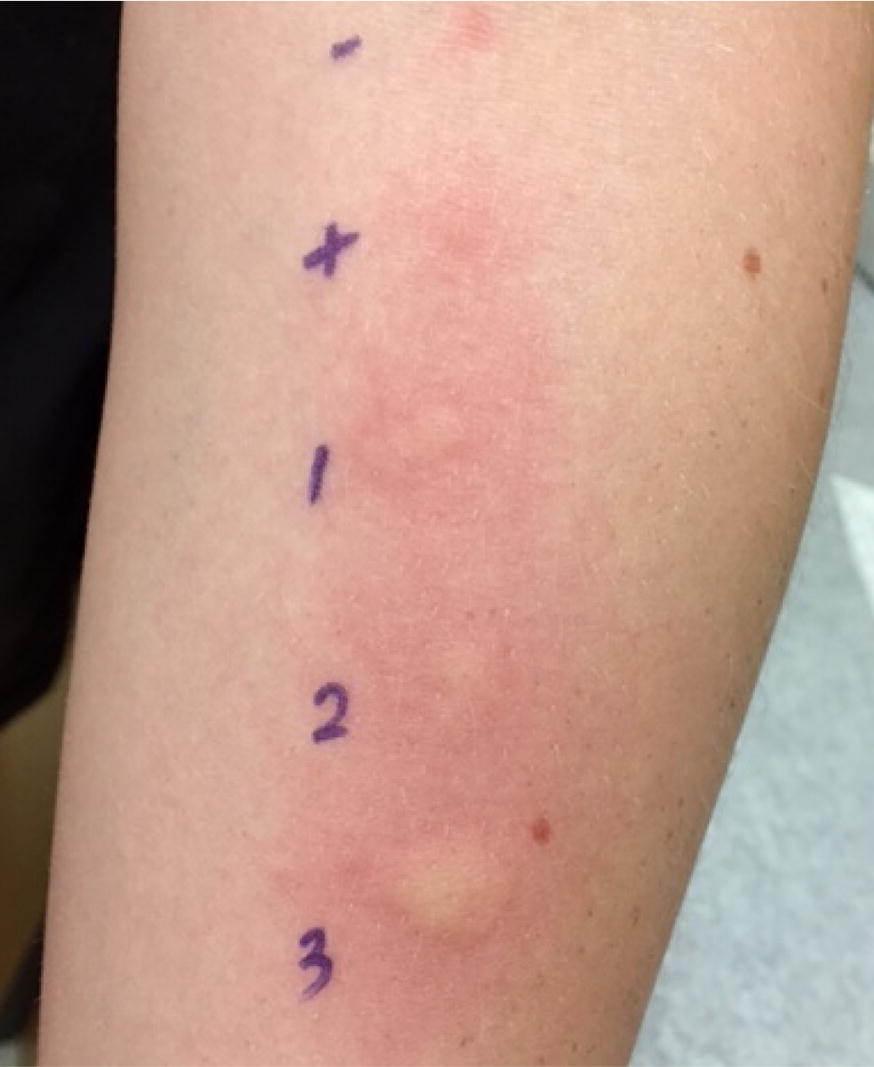
SPT results. 1. Pancake mix containing lupine (10 × 7 millimetre (mm) wheal), 2. Fresh lupine bean (12 × 6 mm wheal), 3. Peanut (14 × 2 mm wheal). Negative and positive (Histamine) controls are shown at the top

We describe a 10-year old Caucasian male with diagnosed peanut and tree-nut allergy, who developed anaphylaxis to lupine flour in May 2017 in Vancouver, Canada. A few minutes after eating a small amount of pancake made with a pre-made mix, he developed oral pruritis, throat tightness, severe stomachache, lightheadedness, cough, hoarse throat, nasal congestion, sneezing, and fatigue. He refused epinephrine but was given cetirizine. The symptoms resolved after 3 h, but he was still unwell the following day. In a conversation between the mother and the allergist, it was suspected that lupine, the second ingredient on the label, was the cause of anaphylaxis, since the patient was eating the other ingredients regularly. He was brought into the BC Children’s Hospital Allergy clinic in June 2017 for skin prick testing to lupine (Fig. 1). Results were consistent with lupine allergy and the patient was counseled to avoid lupine. The mother was counseled on the importance of administering epinephrine for anaphylaxis. In addition, the mother was encouraged by the allergist to report this incident to the food company and government agencies.

Subsequently, the food company performed testing on the pancake mix and confirmed that it did not contain the patient’s known allergens.

After the incident, the patient’s mother became an advocate for patient education regarding cross-reactivity between lupine and peanut, and importance of labeling lupine-containing products to warn families with peanut allergy about lupine. She contacted the Canadian Food Inspection Agency (CFIA), who issued a product recall and public information sheet (see Additional file 1), and Health Canada, who issued “Information for Canadians with peanut allergy concerning lupine”, a message about peanut allergy and exposure to lupine as a food ingredient. The pancake mix company initially recalled the product from stores, but in a letter to the family a few months later, they wrote they would be putting the product back on the shelf with a warning label that lupine is a legume related to peanuts. The company promised to include warning labels for other home-brand lupine-containing products and institute a voluntary labeling system for other companies whose lupine-containing products may be sold in their stores. The mother was interviewed in magazines, e-newsletters, and blogs, aimed at educating parents of children with food allergy.

## Discussion and conclusions

This is the first reported case of an allergic reaction to lupine in Canada. The family had never heard about lupine and its cross-reactivity with peanut, highlighting the need for education of families with peanut allergy. Our patient’s mother contacted many different organizations—the company, CFIA, Health Canada—as there was not a clear reporting mechanism for emerging allergens. She also met with her local Member of Parliament, who drafted a letter to the Federal Minister of Health in support of lupine being recognized as a priority allergen by Health Canada.

Until recently, lupine has not been widely available in Canada, but will likely become more popular because of its purported health benefits [8]. Therefore, more education about lupine is necessary for peanut-allergic Canadians and those who prepare foods for peanut-allergic individuals. Allergists should be aware of lupine cross-reactivity [3, 4] and its emerging presence in the Canadian food supply, and should mention it to their patients when counseling about reading food labels. While it is not required, manufacturers of products containing lupine could consider adding a disclaimer on their product near the ingredient list that “Lupine may trigger allergic reactions in some people with peanut allergy” or similar wording. This case illustrates the need for a simple mechanism for consumers to report emerging allergens to regulatory agencies.

This case also illustrates the need for patient education on the appropriate management of anaphylaxis. The epinephrine autoinjector was not administered because the child refused it, even though the symptoms were unequivocal for anaphylaxis. Since this incident, the patient has experienced anaphylaxis in the allergy clinic during an oral food challenge and administered the autoinjector with the support of the allergist. He commented after this experience that the injection was painless, he felt better immediately, and he would use the autoinjector in the future.

This case provided impetus for a collaborative project between BC Children’s Hospital, the patient’s mother, and Health Canada, who has a high degree of interest in being notified of emerging allergens (e.g. lupine) in Canada as quickly as possible. We are currently in the process of designing a protocol for consumers to report emerging allergens in the Canadian food supply to Health Canada. This protocol will be consumer-driven, as our case illustrates the effectiveness of patient-led reporting. However, confirmation of the reaction by an allergist as IgE-mediated is important, and our protocol will require allergy testing (including an oral food challenge when indicated) and written confirmation from their allergist before consideration by Health Canada as an emerging allergen. To be defined as an emerging allergen would be a first step before addition to the list of priority allergens as defined by Health Canada criteria [9].

## Additional file

**Additional file 1.** Canadian Food Inspection Agency Consumer Advisory-Lupin may cause allergic reactions in peanut allergic consumers.

## Abbreviations

CFIA: Canadian Food Inspection Agency
Mm: millimetres
SPT: skin prick test
