# Supplementary material for: First reported case in Canada of anaphylaxis to lupine in a child with peanut allergy
Source: Allergy Asthma Clin Immunol. 2018 Oct 29;14:64. doi: 10.1186/s13223-018-0303-4 (PMC6205782; doi:10.1186/s13223-018-0303-4)
Supplement: Supplementary file 1 — Additional file 1. Canadian Food Inspection Agency Consumer Advisory-Lupin may cause allergic reactions in peanut allergic consumers. [file 13223_2018_303_MOESM1_ESM.pdf]

Notice

This archive of previously issued food recalls and allergy alerts is provided for reference and research purposes.

Users should note that the products listed in the archive have been subject to removal from the marketplace or appropriate corrective action. Food recalls or allergy alerts are not an indication of the food safety status of products produced at a later date.

Consumer Advisory - Lupin may cause allergic reactions in peanut allergic consumers

Recall / advisory date:

May 12, 2017

Reason for recall / advisory:

Allergen - Other

Hazard classification:

--

Company / Firm:

--

Extent of the distribution:

Consumer

Contents

- Advisory details
- Affected products
- More information

- Media enquiries

Advisory details

Ottawa, May 12, 2017 - Peanut allergic consumers should be aware that lupin can potentially cause allergic reactions. The Canadian Food Inspection Agency is working with Health Canada on preparing information related to the potential risk of lupin for peanut allergic consumers, which will be shared with the public by Health Canada.

In response to reported reactions from consumers, Loblaw Companies Limited has voluntarily recalled PC (President's Choice) Blue Menu brand Buttermilk Protein Pancake Mix and Apple Cinnamon Flavour Protein Pancake Mix from the marketplace. These products contain lupin pulse flour as an ingredient. While the lupin pulse flour was correctly identified on the label, this ingredient could cause a potential reaction in consumers with peanut allergies.

Affected products

Brand Name	Common Name	Size	Code(s) on Product	UPC (Universal Product Code)
**PC (Presidents Choice) Blue Menu**	Buttermilk Protein Pancake Mix	340 g (gram)	All codes	0 60383 18131 4
**PC (Presidents Choice) Blue Menu**	Apple Cinnamon Flavour Protein Pancake Mix	340 g (gram)	All codes	0 60383 18132 1

More information

Loblaw Companies Limited: 1-888-495-5111 or http://www.newswire.ca/news-releases/presidents-choice-recalling-blue-menu-protein-pancake-mixes-due-to-potential-allergen-risk-621909793.html

For more information, consumers and industry can contact the CFIA (Canadian Food Inspection Agency) by filling out the online feedback form.

Media enquiries

CFIA (Canadian Food Inspection Agency) Media Relations

613-773-6600

Share this page

Date modified:

2017-05-12
